# Association between creatinine-to-albumin ratio and mortality in intracerebral hemorrhage: a superior predictive indicator

**DOI:** 10.3389/fneur.2025.1625410

**Published:** 2025-08-05

**Authors:** Yuhong Guo, Qiaoyu You, Peng Wang, Jun Wan, Sen Zhang, Shijie Fan, Yikang Ouyang, Xiang Yuan, Tiangui Li, Cuyubamba Dominguez Jorge Luis, Yu Zhang

**Affiliations:** ^1^Department of Neurosurgery, Shanxi Provincial People’s Hospital, Taiyuan, China; ^2^Center for Evidence-Based Medicine, Affiliated Hospital of Chengdu University, Chengdu, China; ^3^Department of Neurosurgery, West China Hospital, Sichuan University, Chengdu, China; ^4^Department of Neurosurgery, West China Longquan Hospital, Sichuan University, Chengdu, China; ^5^West China Hospital, Institutes for Systems Genetics, Sichuan University, Chengdu, China

**Keywords:** intracerebral hemorrhage, creatinine-to-albumin ratio, mortality, complications, prognostic marker

## Abstract

**Background:**

Intracerebral hemorrhage is a severe and devastating condition with a high mortality rate worldwide. While creatinine and albumin levels have been studied individually, the prognostic value of the creatinine-to-albumin ratio in predicting mortality in intracerebral hemorrhage patients remains underexplored.

**Methods:**

We performed a retrospective cohort study of intracerebral hemorrhage patients from West China Hospital of Sichuan University (December 2010–July 2019) and The First People’s Hospital of Longquanyi District, Chengdu (January 2017–October 2020). Serum biomarker data from blood samples were collected within 24 h of admission. The primary outcome is mortality, while secondary outcomes include renal, infectious, and neurological complications.

**Results:**

A total of 3,521 primary intracerebral hemorrhage patients were included in this study. Based on the Youden Index, 0.30 is the optimal threshold for dichotomizing creatinine-to-albumin ratio. Multivariate analysis showed that patients in higher quartiles of the creatinine-to-albumin ratio had significantly higher in-hospital mortality risks compared to those in the lowest quartile (Q1, reference group) (Q2: aOR 2.38, 95% CI: 1.40–4.03; Q3: aOR 2.87, 95% CI: 1.70–4.84; Q4: aOR 6.11, 95% CI: 3.68–10.15). Similar associations were observed for 30-day, 180-day, and 1-year mortality. Restricted cubic splines further supported this positive dose–response relationship. Receiver operating characteristic analysis showed that the creatinine-to-albumin ratio outperformed the neutrophil-to-lymphocyte ratio and platelet-to-lymphocyte ratio in prognostic performance, especially in predicting in-hospital mortality (AUC = 0.74). Additionally, the dynamic changes in creatinine-to-albumin ratio showed a significant increase in AUC over time (*p* < 0.001 for trend).

**Conclusion:**

Creatinine-to-albumin ratio can serve as an independent and superior predictor of mortality and complications in intracerebral hemorrhage patients. Its prognostic ability could surpass that of commonly used indicators, and its dynamic changes may provide additional valuable insights for prediction. However, further prospective studies are required to confirm its clinical applicability.

## Introduction

Intracerebral haemorrhage (ICH) is the most devastating type of stroke with persistently high mortality and disability rates, making it a significant concern in global public health (1, 2). Although treatment strategies have improved recently, the clinical prognosis of ICH patients remains poor, mainly due to complications such as organ injury, infections, and neurological complications, as well as the failure to promptly identify high-risk patients, leading to higher mortality (3–6). Therefore, timely identification of high-risk patients during the acute phase, coupled with the implementation of effective interventions, can substantially improve survival rates and enhance the quality of life for patients (7, 8).

Renal dysfunction and malnutrition are key factors affecting the prognosis during the acute phase of ICH. Renal dysfunction, such as acute kidney injury, not only triggers a systemic inflammatory response and metabolic disturbances but also affects cerebral hemodynamics, leading to worsened hypertension and neurological damage (9, 10). Meanwhile, the deterioration of nutritional status leads to weakened immune function, increasing the risk of infections (11, 12). These factors can exacerbate brain injury and delay recovery, leading to an increased risk of complications and death.

Peripheral biomarkers, such as the creatinine-to-albumin ratio (CAR), directly integrate renal function and nutritional status, while indirectly reflecting the patient’s inflammatory and immune status, showing prognostic potential in critical conditions. However, its association with mortality in ICH patients remains insufficiently explored. Our study aims to investigate the impact of CAR, based on serum creatinine and albumin, on mortality and complications in ICH patients, evaluate its potential as a prognostic indicator, and compare it with other commonly used prognostic biomarkers.

## Materials and methods

### Study design

This is a multicenter retrospective cohort study targeting ICH patients. We consecutively evaluated the electronic health records of two tertiary hospitals: West China Hospital, Sichuan University (from December 2010 to July 2019), and The First People’s Hospital of Longquanyi District, Chengdu (from January 2017 to October 2020).

The study was reviewed and approved by the institutional review boards of all participating hospitals, including West China Hospital (No. 2021-624) and The First People’s Hospital of Longquanyi District, Chengdu (No. AF-AK-2022010). Informatics staff retrieved data from the electronic health record system, which included medical history, diagnoses, inpatient admission records, discharge records, as well as laboratory and imaging results. All study procedures adhered to the STROBE guidelines and complied with the ethical principles outlined in the Declaration of Helsinki. Due to the retrospective nature of the study, the requirement for informed consent was waived.

### Patient selection

Primary ICH is defined as bleeding within the brain caused by the spontaneous rupture of cerebral blood vessels, in the absence of external trauma or other known structural causes. All patients diagnosed with primary ICH were included in the study, while those diagnosed with secondary ICH, such as bleeding caused by brain aneurysms, cerebrovascular malformations, and head trauma, were excluded. The diagnosis of ICH was confirmed upon admission through computed tomography or magnetic resonance imaging, as well as intraoperative assessments performed by neurosurgeons during hospitalization.

Patients were excluded if they met any of the following criteria: (1) age younger than 18 years; (2) patients whose personal identification number was not found in the electronic health record or whose death record was not found in the Household Registration Administration System; (3) the creatinine and albumin measurements had not been collected within the first 24 h after admission.

### Exposure

The primary exposure variable of this study was CAR, calculated by dividing serum creatinine by serum albumin, both measured from blood samples collected within the first 24 h after admission.

CAR was analyzed using both categorical and continuous variables to assess its association with mortality. First, the optimal cut-off point was determined using Youden Index, categorizing patients into two groups: low CAR group (<0.30) and high CAR group (≥0.30). Second, CAR was stratified into quartiles based on its distribution (Q1: 0.04–0.17, Q2: 0.17–0.22, Q3: 0.22–0.29, Q4: >0.29). Third, CAR was treated as a continuous variable and analyzed per one standard deviation (SD) increase.

### Covariates

We obtained demographic variables (age and sex), lifestyle history (smoking and alcohol use), relevant comorbidities including hypertension, diabetes, and chronic kidney disease, as well as medication history (including statins and anticoagulants) and surgical history (including hematoma evacuation and external ventricular drainage). Additionally, hematoma characteristics (size and location) and GCS scores were also collected.

### Outcomes

The primary outcome of the study was in-hospital mortality, as well as 30-day, 180-day, and 1-year mortality. The secondary outcomes include renal, infectious, and neurological complications. Renal complications include acute kidney injury. Infectious complications include urinary tract infections, pneumonia, bloodstream infections, and intracranial infections. Neurological complications include hydrocephalus and seizures. These complications were predefined based on established clinical criteria.

Acute kidney injury was defined based on KDIGO criteria (13). The diagnosis of all infection-related complications meets the standards set by the Centers for Disease Control and Prevention (14). Hospital-acquired infections are defined as infections that occur more than 48 h after admission during hospitalization. The identification of hydrocephalus and seizures relied on the International Classification of Diseases, Tenth Revision codes and Chinese text. The date of death was ascertained from the Household Registration Administration System, also known as the Chinese Hukou System. According to the law, when a citizen dies in China, their family must notify the household registration authority and cancel the registration within 1 month (15). In 2021, the Seventh National Population Census updated this system, with the National Bureau of Statistics reporting a missing registration rate of 0.05% (16). Therefore, the accuracy of death records in this system is high, and the loss to follow-up in this study is minimal.

### Statistical analysis

All statistical analyses were performed with R version 4.4.1 (The R Foundation for Statistical Computing). Categorical variables were reported as counts (frequencies) and compared using the chi-square test. Continuous variables were presented as means (with standard deviations) and compared using analysis of variance for normally distributed data, or the Wilcoxon rank sum test (Kruskal–Wallis test for more than two categories) for non-normally distributed data. A two-sided *p*-value of less than 0.05 was considered statistically significant. Missing data were estimated using multiple imputation, with five imputed datasets generated through predictive mean matching to reflect the uncertainty of the missing data. By combining the results of all datasets, this method improves accuracy and stability, and has been shown to effectively reduce bias compared to other methods (17).

Logistic regression models were used to adjust for confounding factors. In the univariate analysis, baseline characteristics associated with a *p*-value <0.10 were included in a backward stepwise multivariable logistic regression model. In the multivariable analysis, unadjusted and adjusted odds ratios (ORs) with 95% confidence intervals (CIs) were calculated for each mortality outcome and complication. *E*-value analysis was used to assess the potential impact of unmeasured confounding on the observed effect. Variance inflation factor (VIF) analysis was used to assess the degree of multicollinearity. Additionally, the line chart is used to reflect the trend of CAR across all mortality outcomes. For missing values in the line chart, Last Observation Carried Forward (LOCF) is used to fill left with the previous valid value and right with the next valid value, ensuring no missing values and reflecting the trend of CAR changes. The box plot is then used to visualize the distribution of CAR across all complications.

We also used restricted cubic splines (RCS) to visualize the nonlinear and dose–response relationships between CAR and mortality. To further explore the association within specific ranges of CAR, we defined an interval based on the interquartile range (IQR) of the CAR distribution as (Q1 − 1.5 × IQR, Q3 + 1.5 × IQR) (18). This interval aims to capture the main distribution range of CAR and focus the analysis on the most representative portion of the data.

Furthermore, we constructed receiver operating characteristic (ROC) analysis to compare the discriminatory ability of CAR and seven other serum biomarkers (NLR, PLR, creatinine, albumin, neutrophils, lymphocytes, and platelets) for predicting mortality. The incremental predictive value of CAR when added to the ICH model for mortality was validated by calculating the C-index, Net Reclassification Improvement (NRI), and Integrated Discrimination Improvement (IDI). We further investigated the predictive value of CAR change (the difference between the CAR value on the day of measurement and the CAR value at admission) for mortality, exploring its potential for dynamic monitoring.

Finally, subgroup analyses were conducted to determine whether the association between CAR and mortality varied across different subgroups, and interaction *p*-values were calculated.

## Results

### Patient characteristics

A total of 7,013 adult patients diagnosed with primary ICH at West China Hospital and The First People’s Hospital of Longquanyi District in Chengdu were included in this study (Supplementary Figure S1). Exclusion criteria included 2,485 patients with incorrect or missing personal identification numbers in the electronic health records and 1,007 patients with missing CAR data upon admission. After applying these exclusions, the final analysis included 3,521 ICH patients. Among them, 424 patients (12.0%) died before discharge, 716 patients (20.3%) experienced 30-day mortality, 911 patients (25.9%) experienced 180-day mortality, and 1,031 patients (29.3%) died within 1 year.

The baseline information and characteristics of the patients are shown in Table 1. According to the Youden Index, 0.30 is the optimal cutoff value for distinguishing between the low CAR group (<0.30) and the high CAR group (≥0.30). Patients in the high CAR group were more likely to be male, have a higher prevalence of hypertension, diabetes, chronic kidney disease, subdural hematoma with larger volume, and lower GCS scores. Additionally, they also had a significantly higher risk of mortality. Their in-hospital mortality (29.4% vs. 6.8%, *p* < 0.001), 30-day mortality (41.1% vs. 14.0%, *p* < 0.001), 180-day mortality (50.9% vs. 18.3%, *p* < 0.001), and 1-year mortality (54.3% vs. 21.7%, *p* < 0.001) were all significantly higher compared to the low CAR group. Missing data included hematoma size (12.3%), neutrophils and lymphocytes (6% each), and platelet count (5.9%). All other variables had no missing values.

**Table 1:** Baseline characteristics of the patients.

Characteristics	CAR			*p*
	Overall (*n* = 3,521)	Low group (*n* = 2,701)	High group (*n* = 820)	
Demographics				
Age, years, mean (SD)	57.86 (14.71)	58.01 (14.59)	57.34 (15.12)	0.25
Female, *n* (%)	1,151 (32.7)	996 (36.9)	155 (18.9)	<0.001
Lifestyle history, *n* (%)				
Smoking, *n* (%)	1,124 (31.9)	831 (30.8)	293 (35.7)	0.009
Alcohol use, *n* (%)	1,099 (31.2)	827 (30.6)	272 (33.2)	0.18
Medical history, *n* (%)				
Hypertension	2,524 (71.7)	1,874 (69.4)	650 (79.3)	<0.001
Diabetes	358 (10.2)	240 (8.9)	118 (14.4)	<0.001
Chronic kidney disease	124 (3.5)	5 (0.2)	119 (14.5)	<0.001
Medication history, *n* (%)				
Statins	236 (6.7)	182 (6.7)	54 (6.6)	0.94
Anticoagulants	325 (9.2)	248 (9.2)	77 (9.4)	0.91
Surgical history, *n* (%)				
Hematoma evacuation	1,008 (28.6)	754 (27.9)	254 (31.0)	0.10
External ventricular drainage	234 (6.6)	187 (6.9)	47 (5.7)	0.26
Hematoma characteristics				
Size of hematoma, ml, mean (SD)	23.78 (27.27)	21.74 (25.40)	30.84 (31.94)	<0.001
Infratentorial hematoma, *n* (%)	647 (18.4)	464 (17.2)	183 (22.3)	0.001
Intraventricular hematoma, *n* (%)	846 (24.0)	641 (23.7)	205 (25.0)	0.49
Glasgow Coma Scale, mean (SD)	10.78 (4.12)	11.38 (3.82)	8.77 (4.43)	<0.001
Laboratory tests, mean (SD)				
CAR	0.33 (0.48)	0.20 (0.05)	0.78 (0.85)	<0.001
NLR	11.46 (9.32)	10.76 (8.70)	13.85 (10.85)	<0.001
PLR	186.16 (126.55)	186.51 (124.10)	184.97 (134.57)	0.77
Creatinine, mg/dL	1.18 (1.54)	0.76 (0.19)	2.55 (2.75)	<0.001
Albumin, g/dL	3.78 (0.60)	3.91 (0.52)	3.34 (0.65)	<0.001
Neutrophil, 10^9^/L	8.84 (5.68)	8.55 (5.20)	9.81 (6.98)	<0.001
Lymphocyte, 10^9^/L	1.06 (0.74)	1.09 (0.76)	0.96 (0.66)	<0.001
Platelet, 10^9^/L	154.66 (71.77)	161.25 (72.37)	132.34 (64.97)	<0.001
Outcome, *n* (%)				
In-hospital mortality	424 (12.0)	183 (6.8)	241 (29.4)	<0.001
30-day mortality	716 (20.3)	379 (14.0)	337 (41.1)	<0.001
180-day mortality	911 (25.9)	494 (18.3)	417 (50.9)	<0.001
1-year mortality	1,031 (29.3)	586 (21.7)	445 (54.3)	<0.001

SD, standard deviation; CAR, creatinine-to-albumin ratio; NLR, neutrophil-to-lymphocyte ratio; PLR, platelet-to-lymphocyte ratio.

### Association between CAR and mortality and complications

Logistic regression analysis revealed that the correlation between CAR levels and all mortality outcomes was significant in both unadjusted and adjusted models (Table 2). In the multivariate logistic regression analysis, after adjusting for confounding factors, the high CAR group (≥0.30) exhibited a higher risk of mortality: in-hospital mortality (aOR = 2.96, 95% CI: 2.21–3.97, *p* < 0.001), 30-day mortality (aOR = 2.70, 95% CI: 2.12–3.43, *p* < 0.001), 180-day mortality (aOR = 2.99, 95% CI: 2.39–3.75, *p* < 0.001), and 1-year mortality (aOR = 2.70, 95% CI: 2.17–3.36, *p* < 0.001). The *E*-values for CAR and in-hospital mortality, 30-day mortality, 180-day mortality, and 1-year mortality are 5.75, 5.21, 5.81, and 5.21, respectively. Therefore, the unadjusted factors are less likely to alter these associations. Meanwhile, the maximum VIF value for all variables is 1.18, and the mean is 1.08, so the multicollinearity issue among the independent variables is relatively low.

**Table 2:** Associations between CAR and mortality using logistic regression.

Outcome	Category	CAR level	Unadjusted OR (95% CI)	*p*	Adjusted OR (95% CI)	*p*
In-hospital mortality	Continuous variable	Per SD	1.50 (1.38–1.63)	<0.001	1.47 (1.29–1.67)	<0.001
	Dichotomous variable	≥0.30	5.73 (4.63–7.08)	<0.001	2.96 (2.21–3.97)	<0.001
	Quartile	Q1 (0.04–0.17)	Reference	<0.001^*^	Reference	<0.001^*^
		Q2 (0.17–0.22)	2.15 (1.39–3.34)		2.38 (1.40–4.03)	
		Q3 (0.22–0.29)	2.86 (1.87–4.36)		2.87 (1.70–4.84)	
		Q4 (>0.29)	10.64 (7.22–15.67)		6.11 (3.68–10.15)	
30-day mortality	Continuous variable	Per SD	1.53 (1.41–1.67)	<0.001	1.55 (1.36–1.77)	<0.001
	Dichotomous variable	≥0.30	4.27 (3.58–5.10)	<0.001	2.70 (2.12–3.43)	<0.001
	Quartile	Q1 (0.04–0.17)	Reference	<0.001^*^	Reference	<0.001^*^
		Q2 (0.17–0.22)	1.36 (1.03–1.80)		1.48 (1.06–2.08)	
		Q3 (0.22–0.29)	1.54 (1.17–2.03)		1.48 (1.05–2.07)	
		Q4 (>0.29)	5.23 (4.07–6.71)		3.38 (2.45–4.67)	
180-day mortality	Continuous variable	Per SD	1.68 (1.53–1.85)	<0.001	1.63 (1.43–1.86)	<0.001
	Dichotomous variable	≥0.30	4.62 (3.91–5.47)	<0.001	2.99 (2.39–3.75)	<0.001
	Quartile	Q1 (0.04–0.17)	Reference	<0.001^*^	Reference	<0.001^*^
		Q2 (0.17–0.22)	1.30 (1.01–1.67)		1.35 (1.00–1.83)	
		Q3 (0.22–0.29)	1.62 (1.26–2.07)		1.51 (1.12–2.04)	
		Q4 (>0.29)	5.60 (4.45–7.03)		3.61 (2.70–4.84)	
1-year mortality	Continuous variable	Per SD	1.69 (1.53–1.87)	<0.001	1.64 (1.44–1.88)	<0.001
	Dichotomous variable	≥0.30	4.28 (3.63–5.05)	<0.001	2.70 (2.17–3.36)	<0.001
	Quartile	Q1 (0.04–0.17)	Reference	<0.001^*^	Reference	<0.001^*^
		Q2 (0.17–0.22)	1.75 (1.38–2.20)		1.66 (1.24–2.21)	
		Q3 (0.22–0.29)	1.75 (1.38–2.20)		1.71 (1.28–2.27)	
		Q4 (>0.29)	5.54 (4.44–6.92)		3.71 (2.78–4.94)	

OR, odds ratio; SD, standard deviation; CI, confidence interval; ^*^p for trend. These models were adjusted for the following factors: *In-hospital mortality*: sex, diabetes, chronic kidney disease, statins, hematoma evacuation, external ventricular drainage, hematoma size and location, and Glasgow Coma Scale. *30-day mortality*: age, hypertension, chronic kidney disease, hematoma evacuation, hematoma size and location, and Glasgow Coma Scale. *180-day mortality*: age, hypertension, diabetes, chronic kidney disease, antithrombotic use, hematoma evacuation, external ventricular drainage, hematoma size and location, and Glasgow Coma Scale. *1-year mortality*: age, alcohol use, smoking, diabetes, chronic kidney disease, antithrombotic use, hematoma evacuation, and external ventricular drainage, hematoma size and location and Glasgow Coma Scale.

The quartile stratification of CAR shows that, compared to patients in the lowest quartile (Q1: 0.04–0.17), those in Q2 (0.17–0.22) and Q3 (0.22–0.29) had a significantly higher risk of in-hospital mortality (Q2: aOR = 2.38, 95% CI: 1.40–3.39; Q3: aOR = 2.87, 95% CI: 1.70–4.84), with the highest risk observed in Q4 (>0.29) (aOR = 6.11, 95% CI: 3.68–10.15, *p* < 0.001) (Table 2). Further investigation was conducted using quartile stratification to explore the association between CAR and 30-day mortality, 180-day mortality, and 1-year mortality. These results indicate that the highest CAR levels (Q4 compared to Q1, Q2, and Q3) are significantly associated with the highest mortality rates across all outcomes.

Additionally, the high CAR group (≥0.30) was associated with a higher risk of infectious complications, including hospital-acquired infections (aOR = 1.29, 95% CI: 1.05–1.58, *p* = 0.02), urinary tract infections (aOR = 1.64, 95% CI: 1.23–2.20, *p* < 0.001), and pneumonia (aOR = 1.32, 95% CI: 1.09–1.60, *p* = 0.004) (Table 3). No significant association was found between the high CAR group and intracranial infections (aOR = 0.90, 95% CI: 0.53–1.53, *p* = 0.69). Moreover, the high CAR group was also strongly associated with the occurrence of acute kidney injury, with the probability of developing acute kidney injury remaining eight times higher in the high CAR group compared to the low CAR group after adjusting for confounding factors (aOR = 8.83, 95% CI: 6.99–11.15, *p* < 0.001). However, high CAR levels do not appear to be strongly associated with the occurrence of some neurological complications, such as hydrocephalus (aOR = 0.78, 95% CI: 0.53–1.16, *p* = 0.23) and seizures (aOR = 0.92, 95% CI: 0.50–1.51, *p* = 0.62).

**Table 3:** Associations between high CAR (≥0.30) and complications using logistic regression.

Complication	Unadjusted OR (95% CI)	*p*	Adjusted OR (95% CI)	*p*
Renal complication				
Acute kidney injury	13.80 (11.29–16.87)	<0.001	8.83 (6.99–11.15)	<0.001
Infectious complication				
Hospital-acquired infection	1.83 (1.56–2.14)	<0.001	1.29 (1.05–1.58)	0.01
Urinary tract infection	2.35 (1.84–2.99)	<0.001	1.64 (1.23–2.20)	<0.001
Pneumonia	1.73 (1.48–2.03)	<0.001	1.32 (1.09–1.60)	0.004
Bloodstream infection	1.96 (1.34–2.86)	<0.001	1.26 (0.80–1.98)	0.33
Intracranial infection	1.22 (0.76–1.94)	0.41	0.90 (0.53–1.53)	0.69
Neurological complication				
Hydrocephalus	0.90 (0.63–1.28)	0.54	0.78 (0.53–1.16)	0.23
Seizure	0.92 (0.54–1.57)	0.77	0.87 (0.50–1.51)	0.62

OR, odds ratio; CI, confidence interval. These models were adjusted for the following factors: *Acute kidney injury*: sex, hypertension, diabetes, chronic kidney disease, hematoma evacuation, hematoma size and location, Glasgow Coma Scale. *Hospital-acquired infection*: age, hypertension, diabetes, chronic kidney disease, statins, antithrombotic use, hematoma evacuation, external ventricular drainage, hematoma size and location, Glasgow Coma Scale. *Urinary tract infection*: smoking, hypertension, diabetes, chronic kidney disease, hematoma evacuation, external ventricular drainage, hematoma size and location, Glasgow Coma Scale. *Pneumonia*: age, hypertension, diabetes, statins, antithrombotic use, hematoma evacuation, external ventricular drainage, hematoma size and location, Glasgow Coma Scale. *Bloodstream infection*: sex, hypertension, diabetes, chronic kidney disease, hematoma evacuation, external ventricular drainage, hematoma size and location, Glasgow Coma Scale. *Intracranial infection*: age, antithrombotic use, hematoma evacuation, external ventricular drainage, hematoma size and location, Glasgow Coma Scale. *Hydrocephalus*: external ventricular drainage, hematoma size and location. *Seizure*: hypertension, antithrombotic use, hematoma size and location, Glasgow Coma Scale.

**Figure 1:**
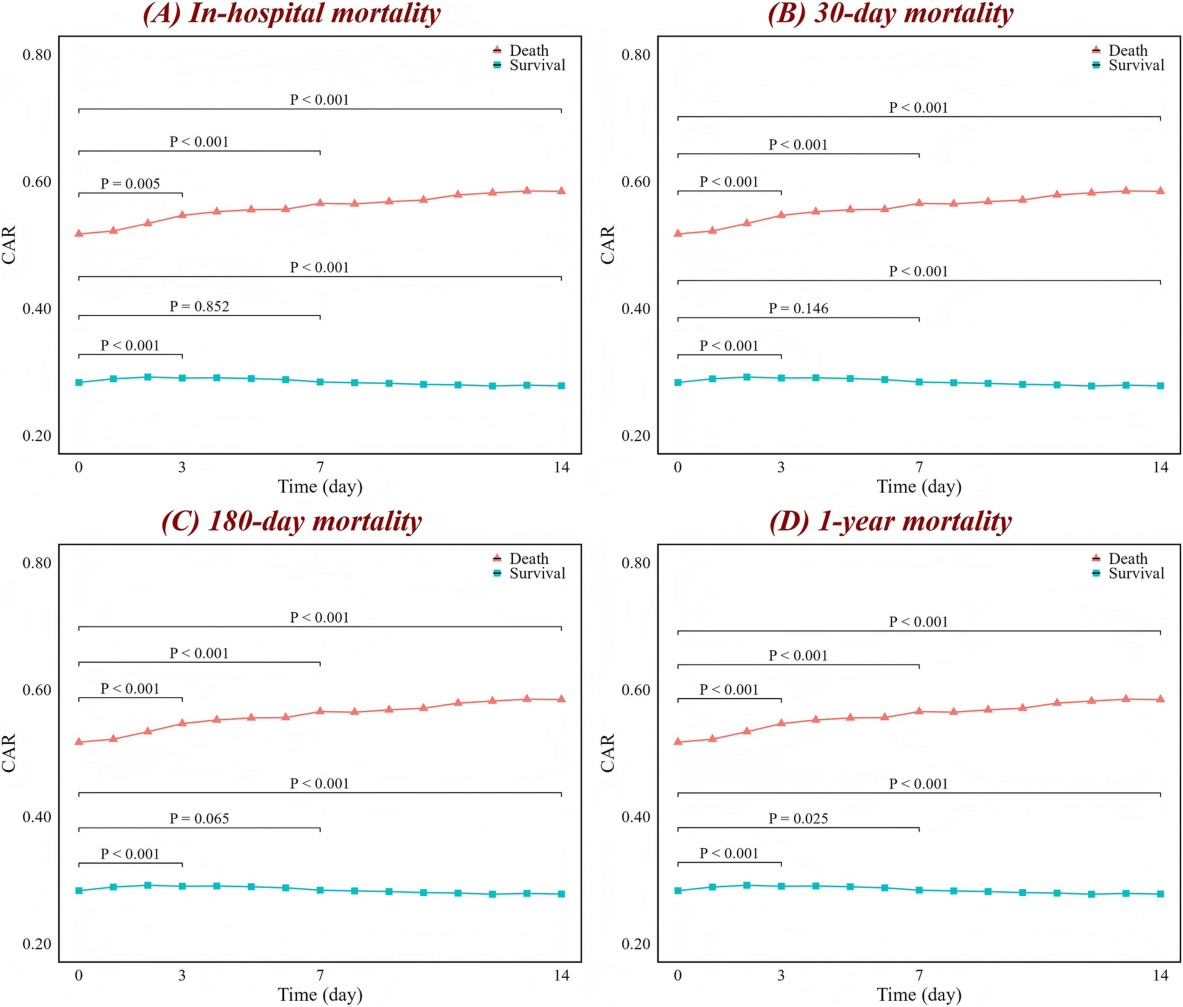
Changes in CAR over time for **(A)** in-hospital, **(B)** 30-day, **(C)** 180-day, and **(D)** 1-year mortality. The red line represents the change in the death group CAR over time, while the blue line represents the survival group. The figure labels the *p*-values for comparisons between day 0 and day 3, day 0 and day 7, and day 0 and day 14, based on the Wilcoxon test. CAR, creatinine-to-albumin ratio.

The association between high CAR values and mortality in ICH patients is shown in Figure 1. Compared to patients with low CAR values, those who died had significantly higher CAR values, which continuously increased as the condition progressed (*p* < 0.001 for trend), while the CAR values of survivors remained relatively stable or even decreased. Similar associations were observed, with patients having infections or acute kidney injury generally showing higher CAR values, while those with hydrocephalus and seizures exhibited little change in CAR levels (Supplementary Figure S2).

**Figure 2:**
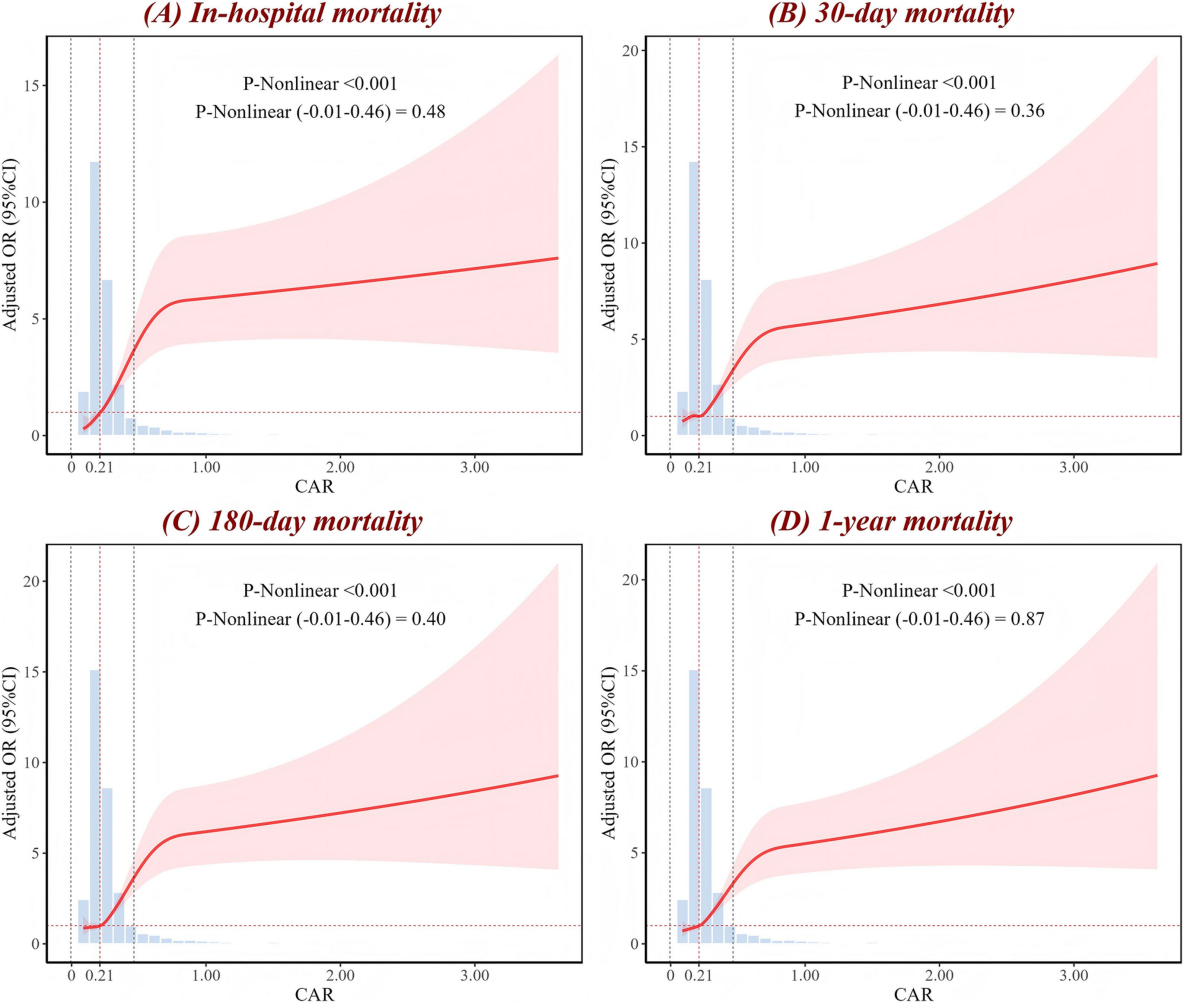
RCS analysis of CAR for **(A)** in-hospital, **(B)** 30-day, **(C)** 180-day, and **(D)** 1-year mortality. The red curve represents the adjusted OR as it changes with CAR, with the shaded area indicating the 95% CI. The figure shows the *p*-value for the overall non-linear association, as well as the *p*-values for the non-linear association within the local range of (−0.01 to 0.46). The intersection of the red dashed line corresponds to an OR of 1. The histogram illustrates the distribution of CAR. OR, odds ratio; CI, confidence interval; CAR, creatinine-to-albumin ratio; RCS, restricted cubic spline.

RCS analysis showed a significant nonlinear association between CAR levels and all mortality outcomes in ICH patients across the full range of CAR values (*p*-nonlinear <0.001) (Figure 2). When the analysis was restricted to the interval of (−0.01, 0.46), the nonlinear relationship transformed into a linear trend (*p*-nonlinear >0.05). However, both across the full range of CAR values and within the specific range, CAR exhibited a positive dose–response relationship with mortality risk. As CAR levels increase, the mortality risk in ICH patients also rises. For in-hospital mortality, when CAR exceeds 0.21, the risk begins to significantly increase. When CAR exceeds 0.30, the mortality rate is approximately 30%, and when CAR exceeds 0.79, the mortality rate reaches as high as 40% (Supplementary Figure S3).

### Promising biomarker of mortality

**Figure 3:**
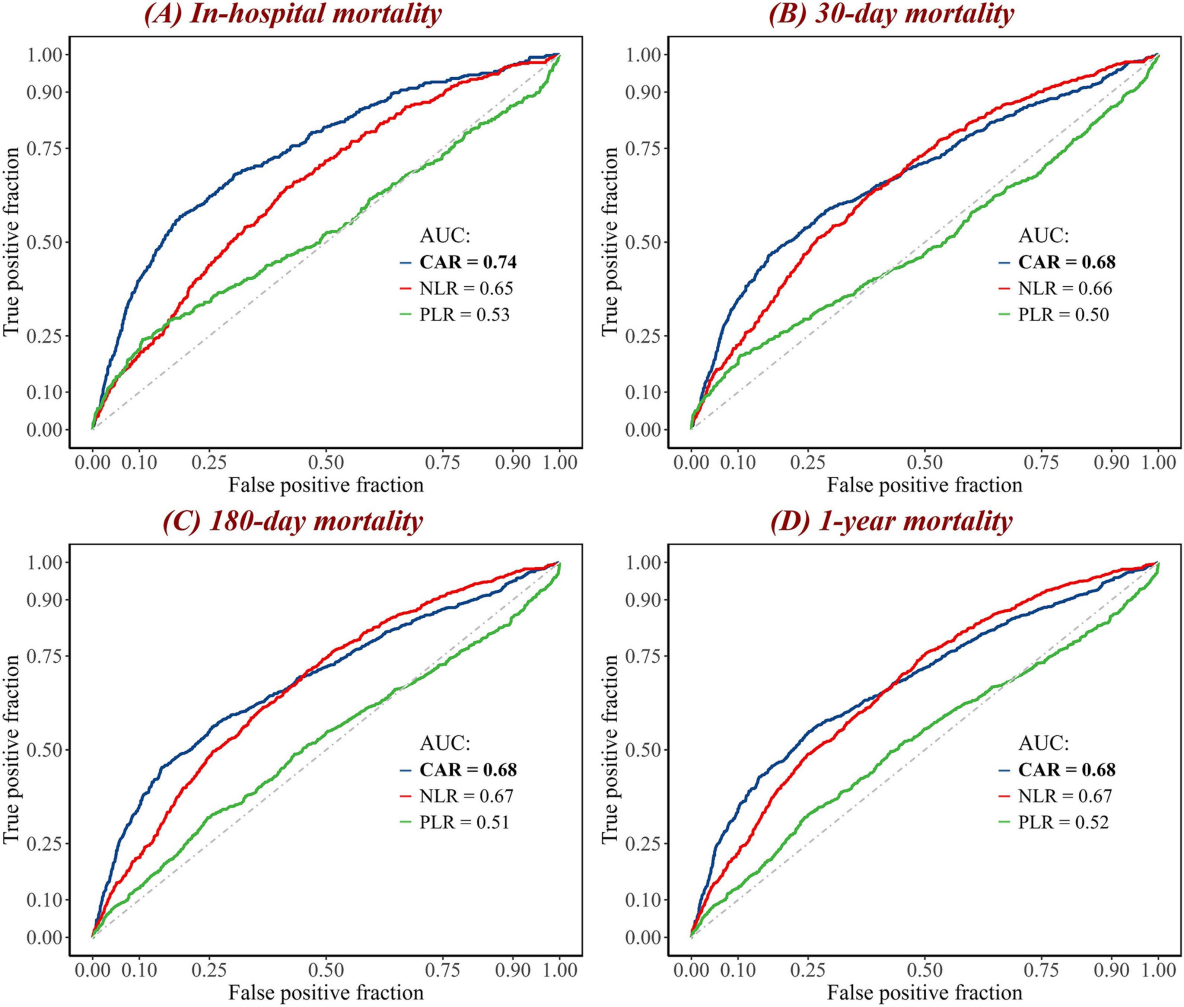
ROC curves of combined biomarkers for **(A)** in-hospital, **(B)** 30-day, **(C)** 180-day, and **(D)** 1-year mortality. The curves represent the performance of three biomarkers in predicting mortality. The blue curve represents the CAR; the red curve represents the NLR; and the green curve represents the PLR. The diagonal gray dashed line is the reference line. The maximum AUC value is highlighted in bold. AUC, area under the curve; CAR, creatinine-to-albumin ratio; NLR, neutrophil-to-lymphocyte ratio; PLR, platelet-to-lymphocyte ratio; ROC, receiver operating characteristic.

ROC curves were generated to evaluate the performance of eight biomarkers (CAR, NLR, PLR, creatinine, albumin, neutrophil, lymphocyte, and platelet) in predicting in-hospital, 30-day, 180-day, and 1-year mortality following patient admission (Figure 3; Supplementary Figure S4). The results showed that, in terms of AUC values, CAR outperformed the aforementioned biomarkers for in-hospital mortality, 30-day mortality, 180-day mortality, and 1-year mortality. For example, compared to NLR (AUC = 0.65) and PLR (AUC = 0.53), CAR had a considerably improved AUC for in-hospital mortality (AUC = 0.74). Furthermore, CAR outperformed the other biomarkers in predicting 30-day mortality (AUC = 0.68), 180-day mortality (AUC = 0.68), and 1-year mortality (AUC = 0.68), demonstrating its consistent and strong predictive ability across these mortality outcomes.

**Figure 4:**
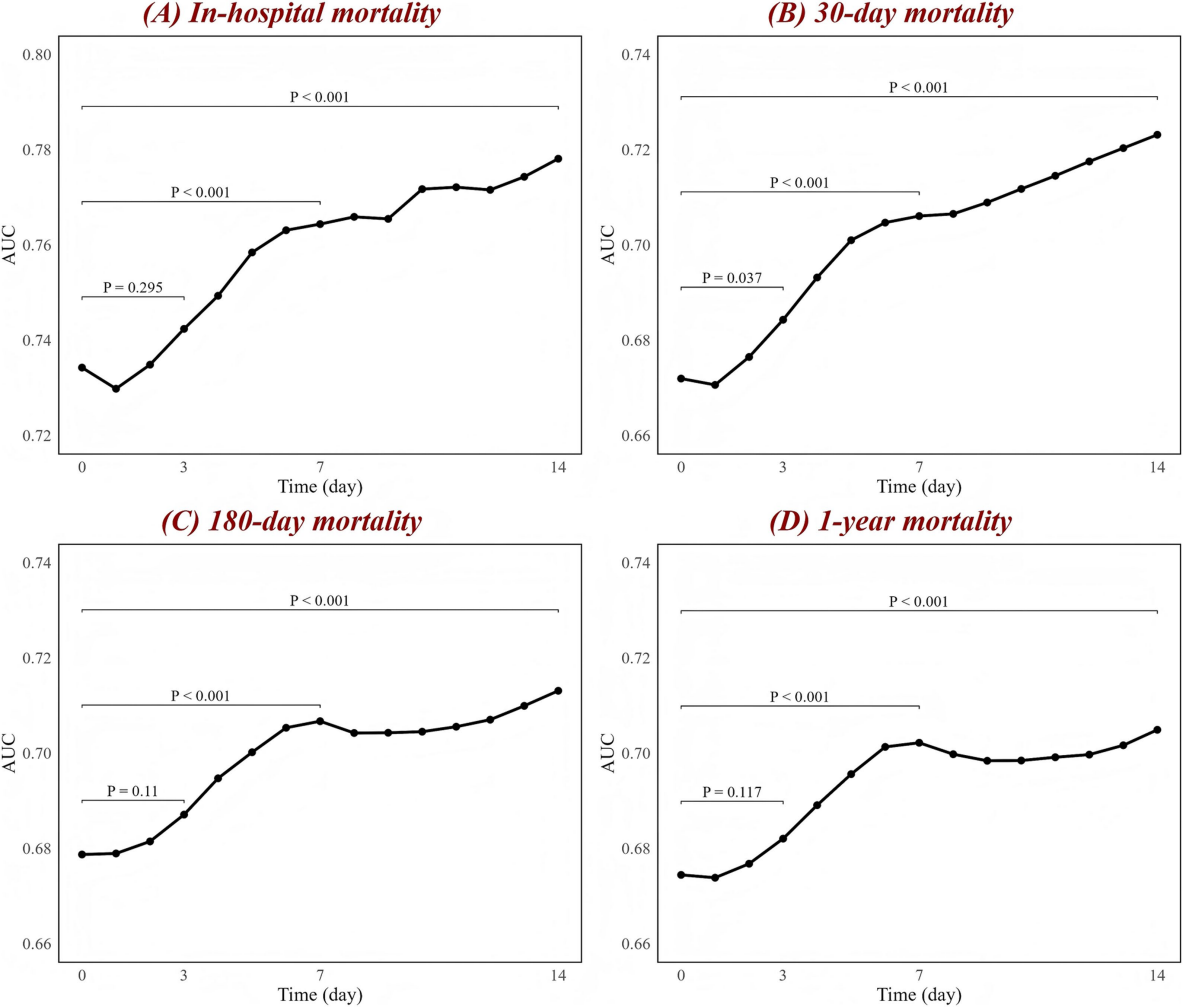
Associations between CAR changes and AUC at different time points for **(A)** in-hospital, **(B)** 30-day, **(C)** 180-day, and **(D)** 1-year mortality. The black line represents the changes in AUC over time. The figure labels the *p*-values for comparisons between day 1 and day 3, day 1 and day 7, and day 1 and day 14, based on the DeLong test. AUC, area under the curve; CAR, creatinine-to-albumin ratio.

To comprehensively compare CAR with other biomarkers, we calculated their C-index, NRI, and IDI based on the ICH model to assess their predictive ability and improvement in model reclassification and discrimination (Table 4; Supplementary Table S1). The results also show that, in predicting all mortality outcomes, CAR was the strongest predictor and had the most significant improvement in the reclassification and discriminatory ability of the ICH model. Other biomarkers, such as NLR, also showed meaningful predictive value. In contrast, several biomarkers, such as PLR, performed poorly. Additionally, the potential predictive value of dynamic changes in CAR for mortality outcomes at different time points is illustrated in Figure 4. Since patients at both hospitals typically undergo CAR measurements on the day of admission, but subsequent measurement time points may vary, this variability was addressed using the LOCF method. The number of patients who underwent CAR measurements at each time point is provided in Supplementary Figure S5. The results reveal that the association between changes in CAR and mortality outcomes progressively strengthened over time. This is evident in the improvement of the AUC, with a significant trend observed (*p* < 0.001 for trend).

**Table 4:** Comparison of NRI and IDI of combined biomarkers in predicting mortality based on the ICH model.

Outcome	Biomarker	C-index (95% CI)	*p*	NRI (95% CI)	P	IDI (95% CI)	*p*
In-hospital mortality	CAR	***0.837 (0.817–0.858)***	<0.001	***0.08 (0.049–0.111)***	<0.001	***0.031 (0.02–0.042)***	<0.001
	NLR	0.823 (0.802–0.844)	0.02	0.005 (−0.02–0.029)	0.70	0.002 (−0.002–0.007)	0.31
	PLR	0.819 (0.796–0.841)	0.46	0.004 (−0.011–0.019)	0.60	0.002 (0–0.003)	0.02
30-day mortality	CAR	***0.819 (0.802–0.837)***	<0.001	***0.033 (0.01–0.056)***	0.005	***0.022 (0.015–0.029)***	<0.001
	NLR	0.812 (0.794–0.83)	0.001	0.029 (0.005–0.053)	0.02	0.005 (0.001–0.009)	0.03
	PLR	0.806 (0.787–0.824)	0.21	0.001 (−0.004–0.006)	0.78	0 (0–0.001)	0.05
180-day mortality	CAR	***0.818 (0.802–0.834)***	<0.001	***0.079 (0.056–0.101)***	<0.001	***0.029 (0.022–0.036)***	<0.001
	NLR	0.806 (0.789–0.822)	0.001	0.012 (−0.008–0.032)	0.24	0.006 (0.002–0.009)	0.002
	PLR	0.8 (0.783–0.817)	0.99	0 (−0.007–0.008)	0.93	0 (0–0.001)	0.11
1-year mortality	CAR	***0.818 (0.802–0.833)***	<0.001	***0.076 (0.056–0.097)***	<0.001	***0.028 (0.022–0.035)***	<0.001
	NLR	0.807 (0.791–0.823)	<0.001	0.042 (0.023–0.061)	<0.001	0.009 (0.005–0.013)	<0.001
	PLR	0.801 (0.785–0.818)	0.62	−0.001 (−0.004–0.002)	0.48	0 (0–0)	0.36

NRI, net reclassification improvement; IDI, integrated discrimination improvement; ICH, intracerebral hemorrhage; CI, confidence interval; CAR, creatinine-to-albumin ratio; NLR, neutrophil-to-lymphocyte ratio; PLR, platelet-to-lymphocyte ratio. The bold values represent the maximum values.

Finally, subgroup analyses showed that CAR was significantly associated with all mortality outcomes across most subgroups, with no highly significant interaction effects (*p* > 0.05) in most subgroups (Supplementary Figures S6–S9). However, the interaction term between CAR and sex subgroups is statistically significant in all mortality outcomes (*p* < 0.05).

## Discussion

We conducted a comprehensive and systematic study on the impact of CAR on adverse outcomes in ICH patients. Our study has made several important findings. First, CAR demonstrates a significant nonlinear dose–response relationship with all mortality outcomes, with a linear trend observed in certain CAR intervals. Second, we revealed that high CAR may be associated with various adverse outcomes, including hospital-acquired infections such as urinary tract infections and pneumonia, as well as acute kidney injury, which could contribute to the increased mortality. Third, compared to other serum biomarkers (such as NLR and PLR), CAR may have a stronger association with mortality outcomes in ICH patients and has shown preliminary potential as a dynamic monitoring tool.

### Comparison with other studies

Previous studies have found that commonly used prognostic biomarkers such as NLR and PLR are valuable in predicting outcomes in patients with ICH (19). However, our study demonstrates that CAR has stronger discriminatory ability than these indicators in predicting mortality in ICH patients (e.g., the AUC for in-hospital mortality is 0.74 vs. 0.64 and 0.53). Even after adjusting for potential confounders in multivariable analyses, CAR retained its independent predictive value, highlighting its robust and consistent performance. Our findings are also consistent with those observed in patients with severe conditions, such as those with sepsis and acute pancreatitis (20, 21). The established CAR threshold of 0.30 is similar to the threshold identified in other studies of patients in critical care, such as those undergoing cardiac surgery (0.31) (22).

### Mechanism

Various mechanisms may explain the association between CAR and mortality risk in ICH patients, as well as its potential superior predictive ability over NLR and PLR in biological terms. First, elevated serum creatinine levels usually indicate renal insufficiency (9). Kidney damage can lead to systemic inflammation, which in turn disrupts the blood–brain barrier (20). Additionally, renal injury is closely associated with other physiological pathways, such as metabolic disorders and coagulation abnormalities (23, 24). Second, serum albumin is an independent predictor of prognosis in patients with ICH (11). Patients with ICH often have lower serum albumin levels, reflecting poor nutritional status and immune function, which increases the risk of complications such as infections (11, 12). Moreover, albumin reflects changes in plasma osmotic pressure, physiological stress, and the inflammatory state associated with nutritional depletion (25, 26). Therefore, in ICH patients, the sustained elevation of CAR levels may be closely associated with the worsening of renal function, malnutrition, or the further exacerbation of systemic inflammatory responses. These factors can intensify brain injury and collectively lead to patient death or severe consequences.

Composite indicators such as NLR and PLR primarily focus on the inflammatory status and immune response in ICH patients. Renal function and nutritional status are also independent prognostic factors for ICH patients. CAR indirectly reflects inflammation and immune response, with a focus on integrating renal function (via serum creatinine) and nutritional status (via serum albumin). Therefore, CAR may provide a more comprehensive assessment and be more strongly associated with the mortality risk and incidence of complications in ICH patients.

### Strengths and limitations

The strength of our study lies in being the first to investigate the relationship between CAR and mortality in patients with ICH, and in the large-scale data collected from two tertiary hospitals.

Nevertheless, this study has several limitations. First, it is a retrospective observational analysis. Although this design helps identify the association between CAR and mortality in ICH patients, it does not allow for definitive conclusions regarding causality, and prospective studies are needed to validate our results. Second, this study was conducted in two tertiary hospitals in Sichuan Province. Although data quality was ensured, the patients were primarily of Asian descent, and the cultural and socioeconomic background of Sichuan may differ from other regions. Additionally, the medical resources available in tertiary hospitals differ from those in primary hospitals. Therefore, the results may be influenced by racial, geographical, and healthcare system differences. Future studies should further validate the applicability of CAR in different racial, regional, and healthcare settings. Third, we have only conducted a preliminary analysis of the relationship between dynamic changes in CAR and mortality, and further research is needed to confirm its association with adverse outcomes. In summary, the lack of longitudinal analysis and the restricted cohort limit the generalizability and applicability of our findings. Finally, although serum creatinine measurement is simple, rapid, and easy to perform, it is influenced by factors such as muscle mass, gender, protein intake, diet, and laboratory testing, which limits its accuracy. Future research should consider using more precise indicators like glomerular filtration rate to more accurately reflect renal function.

## Conclusion

In ICH patients, CAR may serve as an independent predictor of mortality. The findings from our study may equip healthcare providers with additional tools for promptly diagnosing ICH patients, assessing disease severity, and treating individuals with poor prognoses. To support the prognostic utility of CAR in ICH patients, large-sample multicenter prospective studies are required.

## Data Availability

The original contributions presented in the study are included in the article/Supplementary material, further inquiries can be directed to the corresponding author.
