# Supplementary material for: Association between creatinine-to-albumin ratio and mortality in intracerebral hemorrhage: a superior predictive indicator
Source: Front Neurol. 2025 Aug 5;16:1625410. doi: 10.3389/fneur.2025.1625410 (PMC12365379; doi:10.3389/fneur.2025.1625410)
Supplement: Supplementary file 1 [file Data_Sheet_1.docx]

**Table S1 Comparison of NRI and IDI of Individual Biomarkers in Predicting Mortality Based on the ICH Model**

| Outcome | Biomarkers | C-index  (95% CI) | P | NRI  (95% CI) | P | IDI  (95% CI) | P |
| --- | --- | --- | --- | --- | --- | --- | --- |
| In-hospital  mortality | Creatinine | 0.835 (0.814-0.856) | <0.001 | 0.073 (0.043-0.103) | <0.001 | 0.024 (0.016-0.033) | <0.001 |
|  | Albumin | 0.827 (0.806-0.848) | 0.004 | 0.031 (0.002-0.06) | 0.04 | 0.009 (0.003-0.016) | 0.003 |
|  | Neutrophil | 0.821 (0.799-0.843) | 0.15 | 0.007 (-0.015-0.028) | 0.54 | 0.003 (-0.001-0.006) | 0.18 |
|  | Lymphocyte | 0.819 (0.797-0.841) | 0.74 | -0.007 (-0.018-0.004) | 0.23 | 0.001 (0-0.002) | 0.04 |
|  | Platelet | 0.829 (0.809-0.85) | 0.008 | 0.028 (-0.006-0.063) | 0.10 | 0.016 (0.009-0.023) | <0.001 |
| 30-day  mortality | Creatinine | 0.818 (0.8-0.836) | <0.001 | *0.037 (0.015-0.058)* | <0.001 | 0.02 (0.014-0.027) | <0.001 |
|  | Albumin | 0.809 (0.791-0.827) | 0.01 | 0.001 (-0.017-0.018) | 0.94 | 0.001 (-0.001-0.003) | 0.40 |
|  | Neutrophil | 0.811 (0.793-0.829) | 0.03 | 0.017 (-0.004-0.038) | 0.11 | 0.008 (0.003-0.014) | 0.001 |
|  | Lymphocyte | 0.806 (0.788-0.824) | 0.92 | 0.006 (-0.004-0.016) | 0.23 | 0.001 (0-0.002) | 0.05 |
|  | Platelet | 0.812 (0.794-0.829) | 0.02 | -0.002 (-0.026-0.022) | 0.86 | 0.008 (0.004-0.013) | <0.001 |
| 180-day  mortality | Creatinine | 0.816 (0.8-0.832) | <0.001 | 0.07 (0.048-0.091) | <0.001 | 0.027 (0.02-0.033) | <0.001 |
|  | Albumin | 0.805 (0.789-0.822) | 0.001 | 0.012 (-0.008-0.031) | 0.24 | 0.003 (0-0.006) | 0.03 |
|  | Neutrophil | 0.805 (0.788-0.822) | 0.01 | 0.013 (-0.006-0.033) | 0.17 | 0.008 (0.004-0.013) | <0.001 |
|  | Lymphocyte | 0.8 (0.783-0.817) | 0.11 | 0 (0-0) | NA | 0 (0-0) | 0.49 |
|  | Platelet | 0.806 (0.789-0.822) | 0.006 | 0.016 (-0.005-0.038) | 0.13 | 0.008 (0.005-0.012) | <0.001 |
| 1-year  mortality | Creatinine | 0.815 (0.8-0.831) | <0.001 | 0.066 (0.046-0.086) | <0.001 | 0.025 (0.019-0.032) | <0.001 |
|  | Albumin | 0.807 (0.791-0.823) | <0.001 | 0.031 (0.011-0.052) | 0.002 | 0.006 (0.003-0.01) | <0.001 |
|  | Neutrophil | 0.805 (0.789-0.822) | 0.01 | 0.021 (0.003-0.039) | 0.02 | 0.008 (0.004-0.011) | <0.001 |
|  | Lymphocyte | 0.801 (0.785-0.818) | 0.49 | 0.003 (-0.004-0.009) | 0.43 | 0 (0-0.001) | 0.69 |
|  | Platelet | 0.807 (0.791-0.824) | <0.001 | 0.031 (0.011-0.051) | 0.002 | 0.009 (0.005-0.013) | <0.001 |

NRI, net reclassification improvement; IDI, integrated discrimination improvement; ICH, intracerebral hemorrhage; CI, confidence interval

**Figure S1 Flow chart of enrollment**


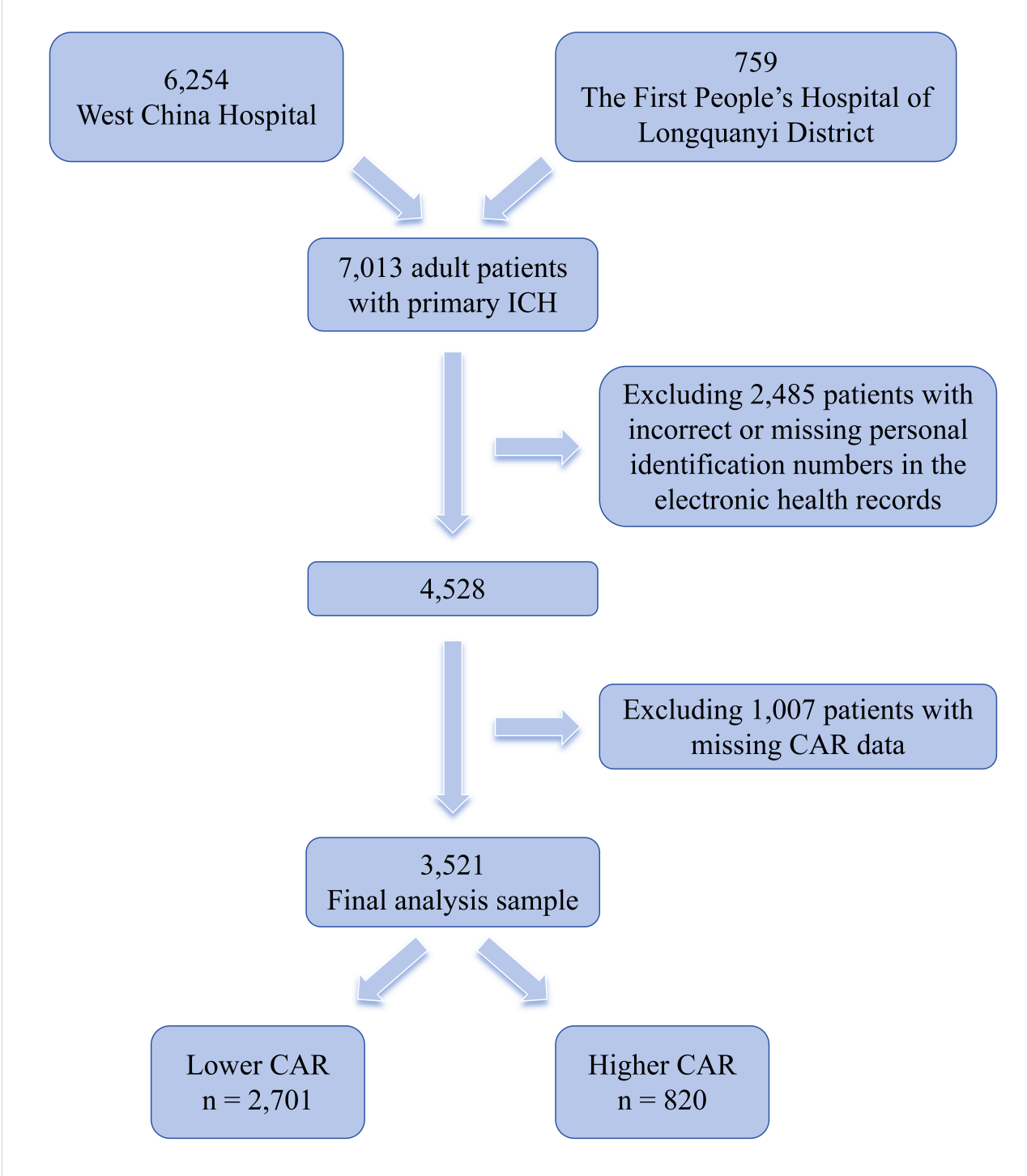


**Figure S2 Boxplot of the Association between CAR and Complications**

**
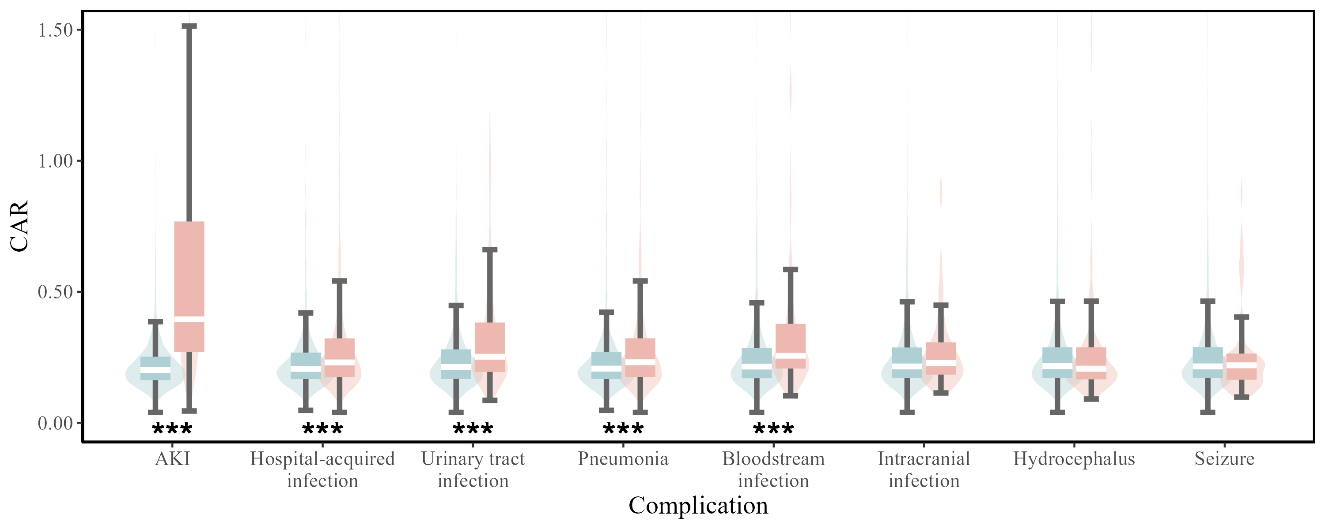
**

**Figure S3 Association Between CAR Threshold and Mortality for (A) In-Hospital, (B) 30-Day, (C) 180-Day, and (D) 1-Year**


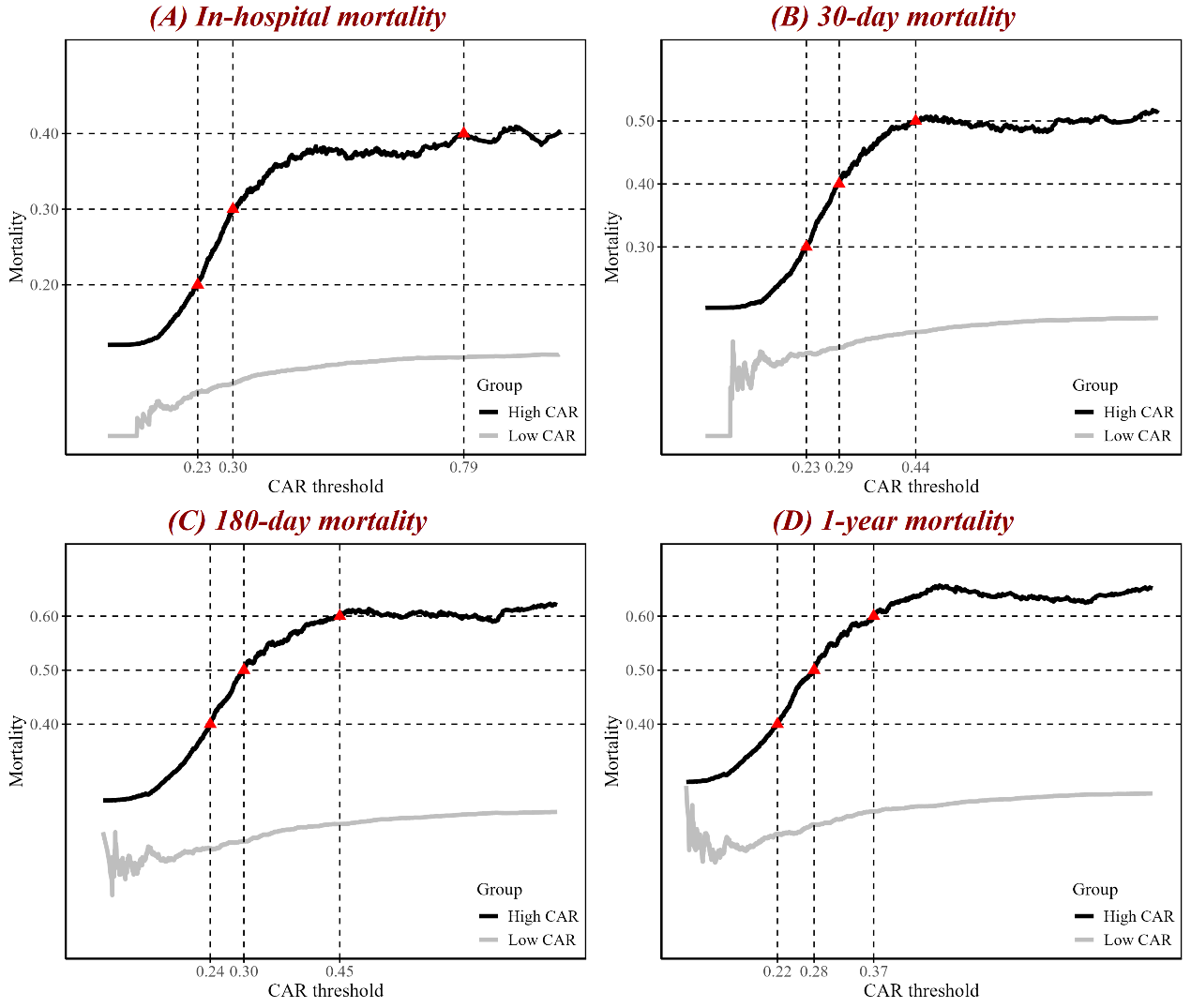


**Figure S4 Receiver Operating Characteristic Curves of Individual Biomarkers for (A) In-Hospital, (B) 30-Day, (C) 180-Day, and (D) 1-Year Mortality**


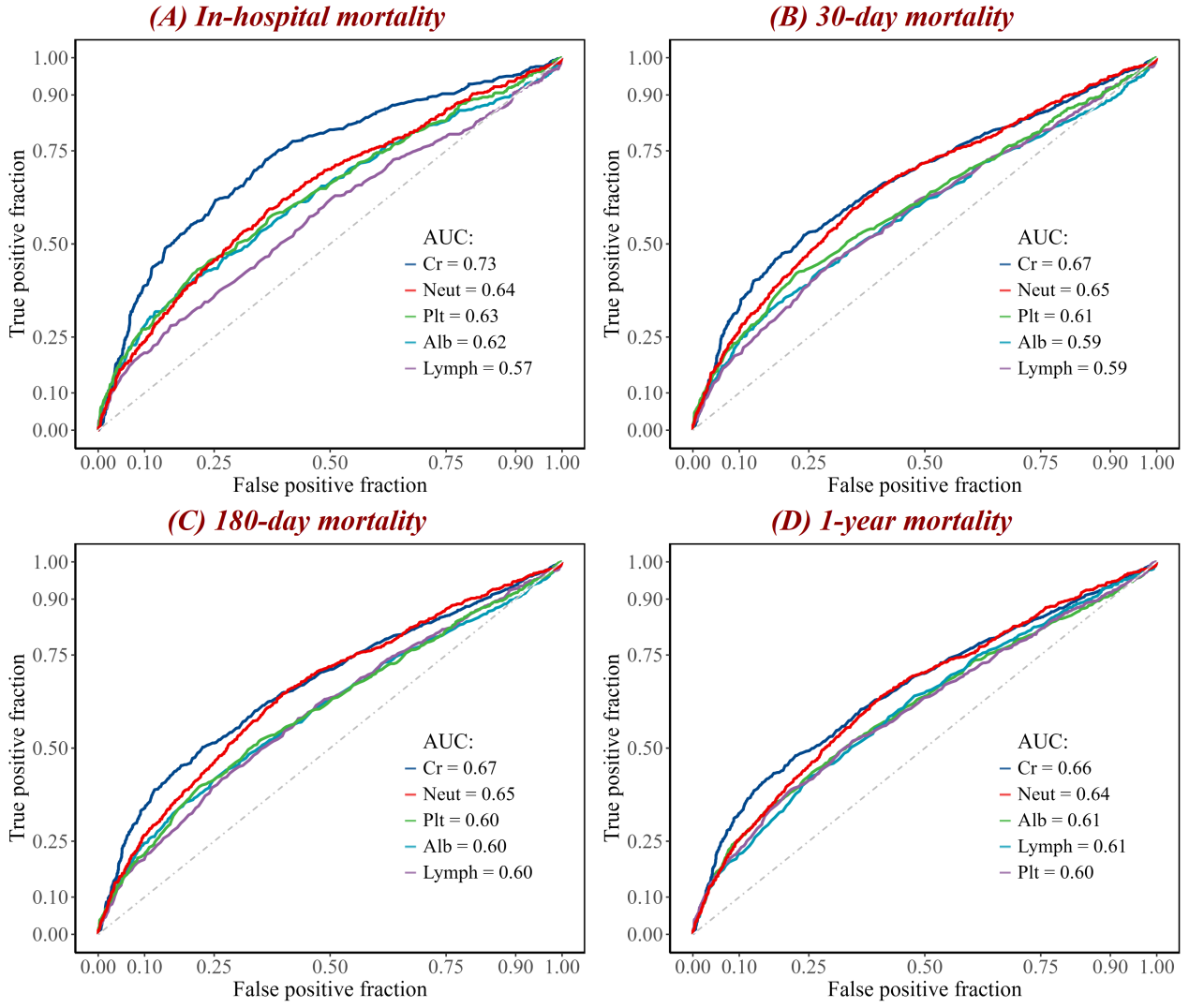


**Figure S5 Number of Patients Undergoing CAR Measurements at Different Time Points**

**
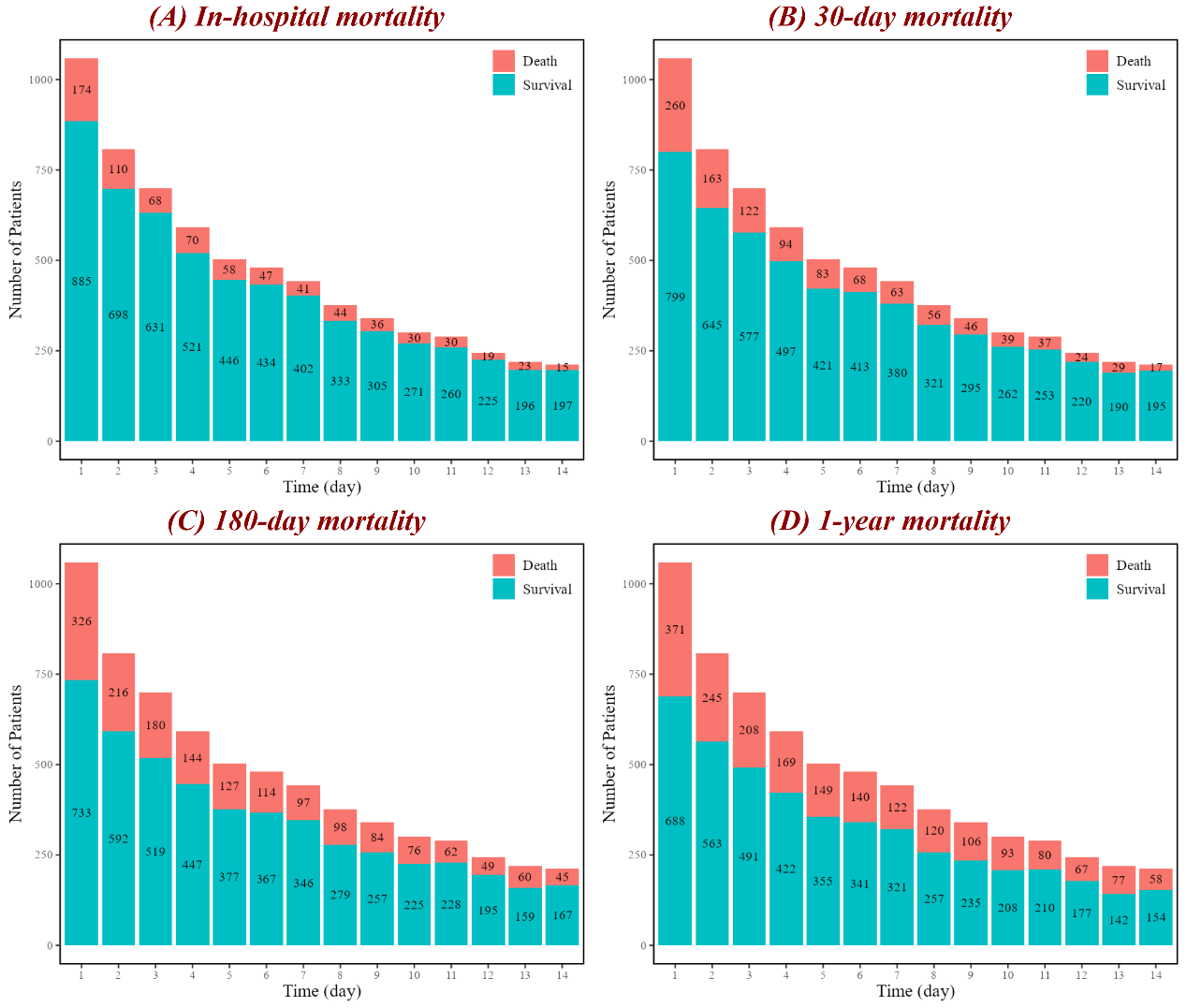
**

**Figure S6 Subgroup Analysis of CAR for In-Hospital Mortality**

**
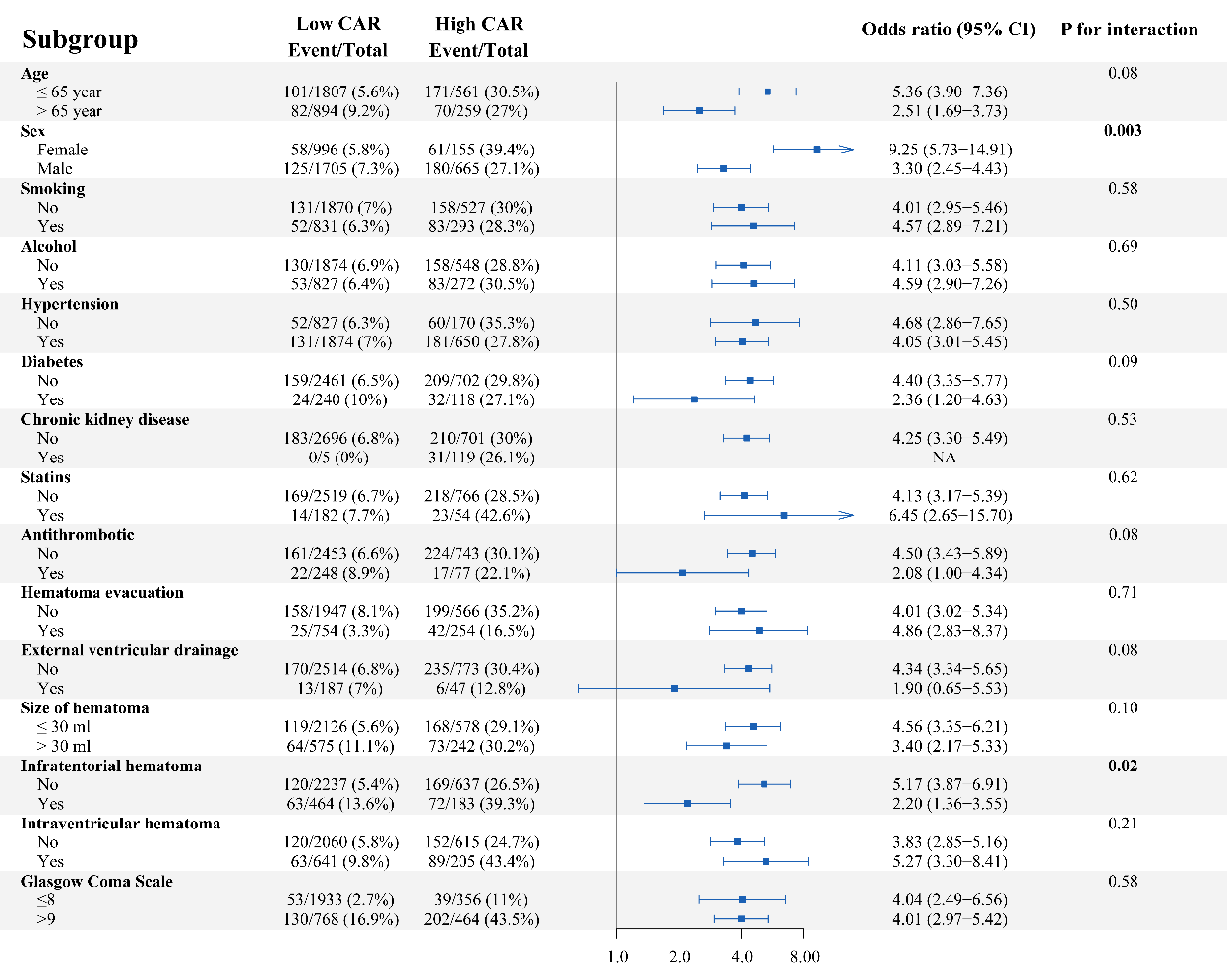
**

**Figure S7 Subgroup Analysis of CAR for 30-Day Mortality**

**
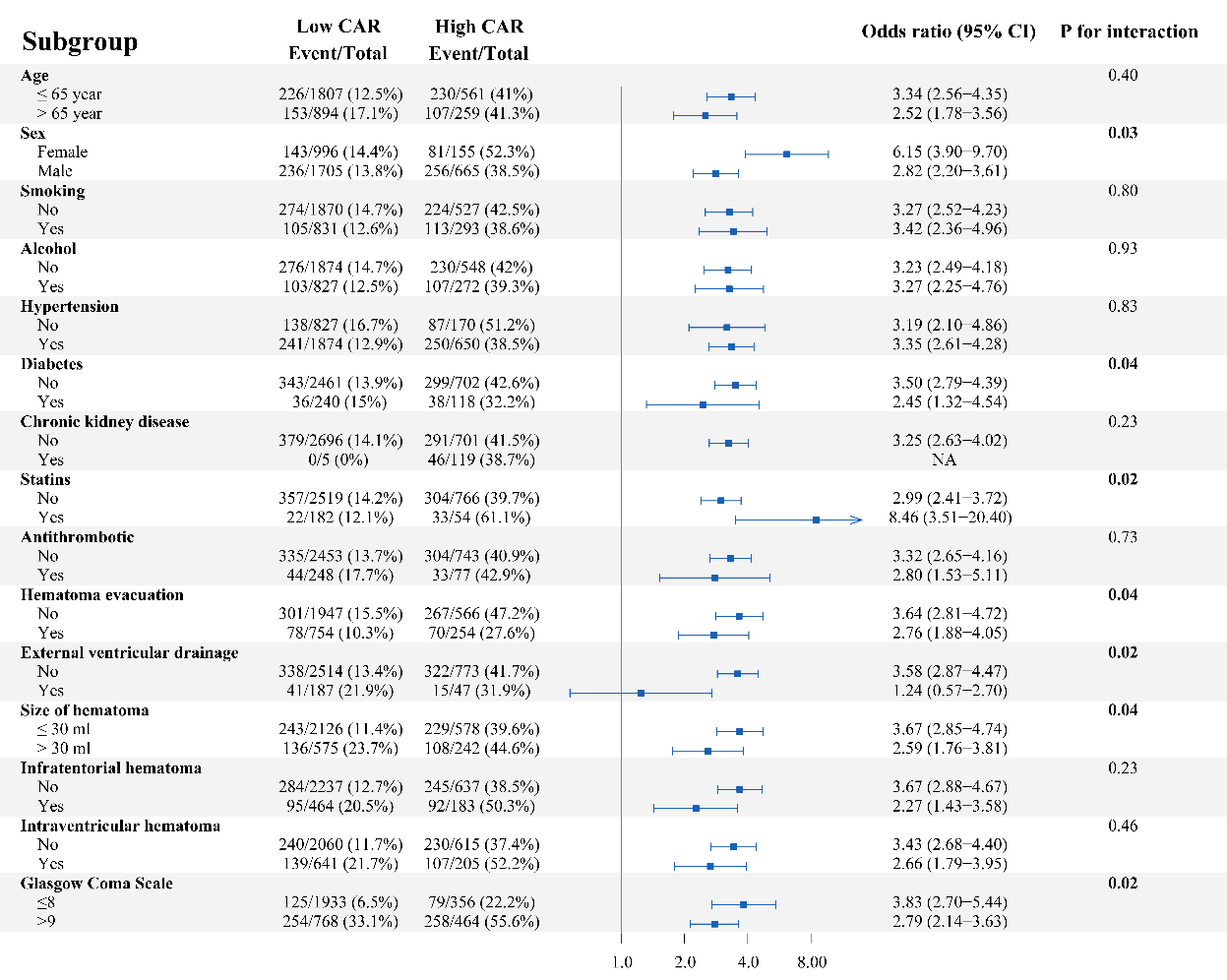
**

**Figure S8 Subgroup Analysis of CAR for 180-Day Mortality**

**
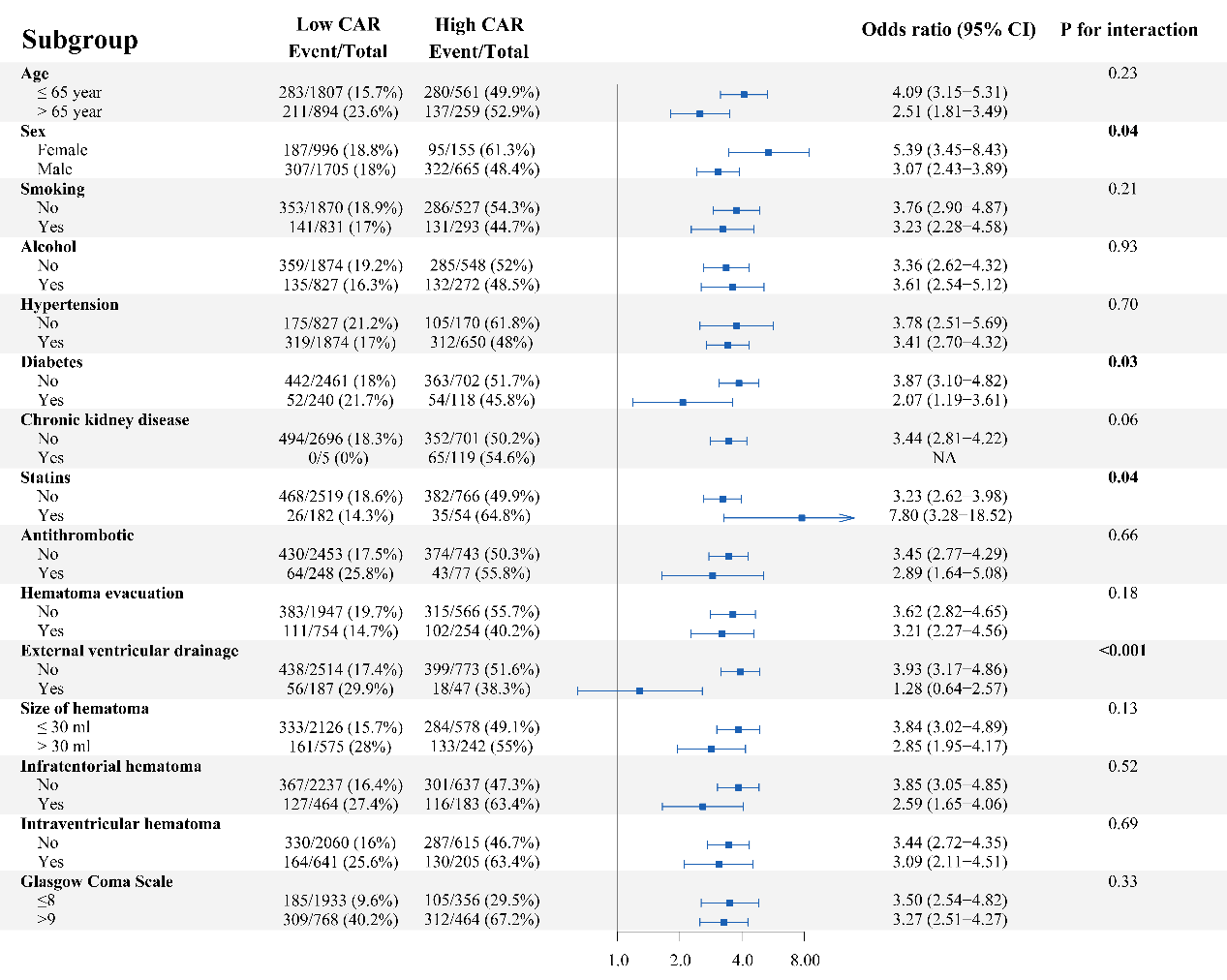
**

**Figure S9 Subgroup Analysis of CAR for 1-Year Mortality**

**
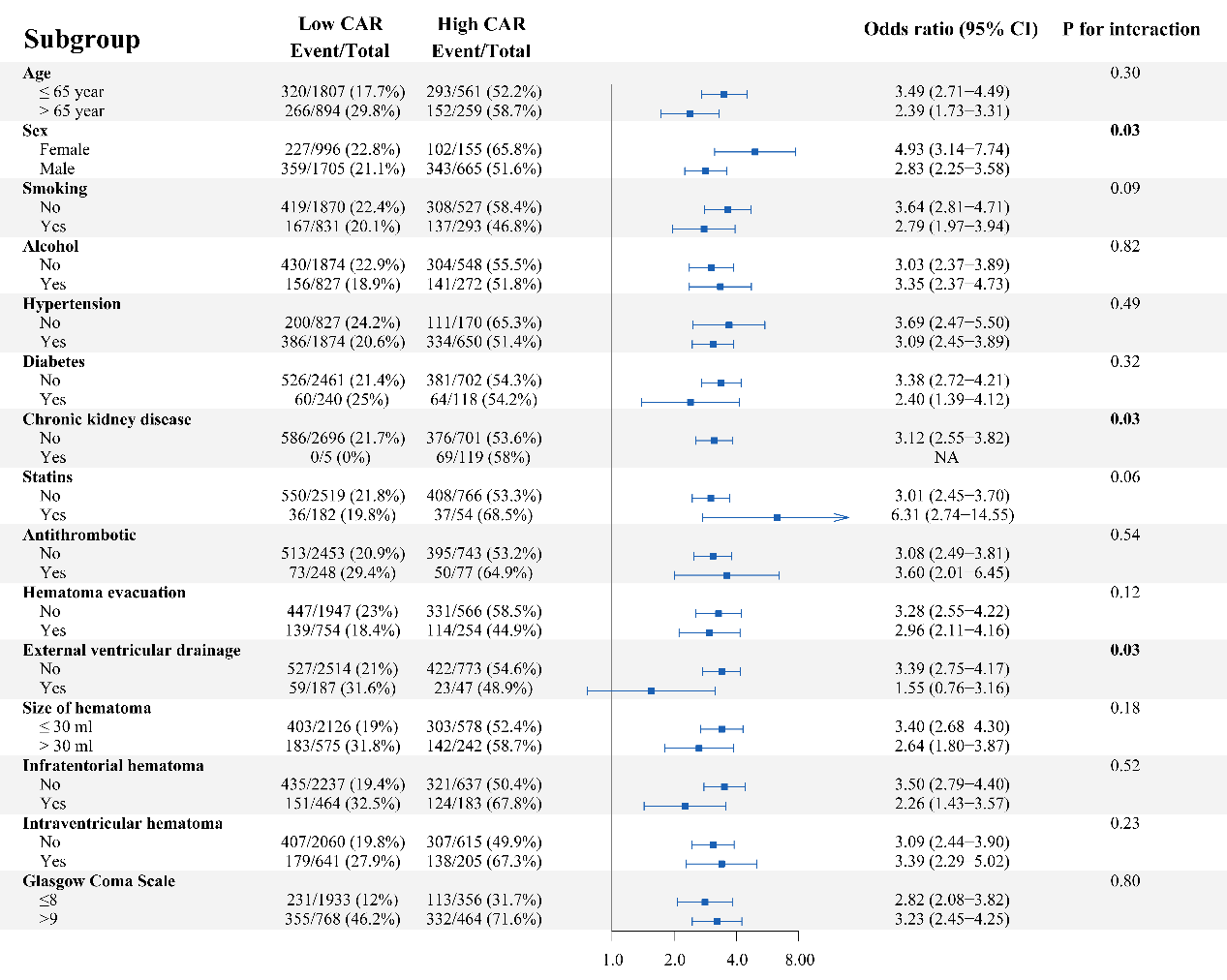
**
